# Light at the end of the tunnel? Follow-up of cardiopulmonary function in children with post-COVID-19

**DOI:** 10.1007/s00431-025-06245-y

**Published:** 2025-06-10

**Authors:** Annika Weigelt, Gunay Akhundova, Roman Raming, Jan-Philipp Tratzky, Adrian P. Regensburger, Calvin Kraus, Wolfgang Waellisch, Regina Trollmann, Joachim Woelfle, Sven Dittrich, Rafael Heiss, Ferdinand Knieling, Isabelle Schoeffl

**Affiliations:** 1 Department of Pediatric Cardiology, University Hospital Erlangen, Friedrich-Alexander-Universität Erlangen-Nürnberg, Loschgestrasse 15, 91054 Erlangen, Germany; 2 Department of Pediatrics, University Hospital Erlangen, Friedrich-Alexander-Universität Erlangen-Nürnberg, Loschgestrasse 15, 91054 Erlangen, Germany; 3 Institute of Radiology, University Hospital Erlangen, Friedrich-Alexander-Universität Erlangen-Nürnberg, Maximiliansplatz 3, 91054 Erlangen, Germany; 4 School of Clinical and Applied Sciences, Leeds Beckett University, Leeds, LS13HE UK

**Keywords:** $\dot{V}{O}_{2}peak$, Cardiopulmonary exercise testing, Post-COVID, Fitness, Exercise capacity, Physical activity

## Abstract

Few studies have examined post-COVID-19 sequelae in children, particularly regarding cardiopulmonary capacity. Longitudinal data are especially scarce. This study aimed to retest pediatric patients previously assessed in a cross-sectional design. In this longitudinal study, children meeting post-COVID-19 criteria and an age- and sex-matched control group underwent cardiopulmonary exercise testing at baseline and after 6 months. Thirteen of 20 post-COVID-19 children (mean age: 13.6 ± 2.6 years, 48% female) and 23 of 28 controls (mean age: 11.9 ± 3.1 years, 62% female) completed follow-up testing. All participants completed a maximal treadmill test. No significant differences were found in peak oxygen uptake ($\dot{V}{O}_{2}peak$ 39.5 ± 11.0 ml/kg/min vs. 45.5 ± 8.4 ml/kg/min; *p* = 0.101). Over 6 months, cardiopulmonary performance improved significantly across all subjects. Subgroup analysis showed improvements in both groups, although changes were not statistically significant. Oxygen pulse also proved to be significantly higher and the half-time recovery of $\dot{V}{O}_{2}$ proved to be significantly longer after 6 months which was true for the overall group but not for the subgroups. *Conclusion*:This is the first longitudinal study to reassess cardiopulmonary capacity in children with post-COVID-19. The initially reduced $\dot{V}{O}_{2}peak$ normalized, and all children showed improved cardiopulmonary capacity after 6 months. The primary improvement was observed in the O_2_ pulse, a surrogate marker of stroke volume and, by extension, cardiac output. This finding suggests an enhancement in cardiovascular performance, reflecting improved central hemodynamic in all children 6 months after the pandemic. Deconditioning thus remains a plausible cause for the post-COVID-19 symptoms. *Trail registration*: ClinicalTrials.gov Identifier: NCT05445531.

**Table:** 

What is Known: • Children with post-COVID-19 (PASC) may exhibit reduced cardiopulmonary function (V̇O2 peak). Fatigue and exercise intolerance are common but poorly understood and objectified. • Previous studies have provided valuable cross-sectional insights but have yet to include longitudinal follow-up data.	
What is New: • First longitudinal CPET-based study reassessing children with PASC after 6 months. • Cardiopulmonary performance, including V̇O2 peak and O2 pulse, improved significantly over time, probably due to reversible deconditioning rather than organ damage.

What is Known:

• Children with post-COVID-19 (PASC) may exhibit reduced cardiopulmonary function (V̇O2 peak). Fatigue and exercise intolerance are common but poorly understood and objectified.

• Previous studies have provided valuable cross-sectional insights but have yet to include longitudinal follow-up data.

What is New:

• First longitudinal CPET-based study reassessing children with PASC after 6 months.

• Cardiopulmonary performance, including V̇O2 peak and O2 pulse, improved significantly over time, probably due to reversible deconditioning rather than organ damage.

## Introduction

Over the course of the coronavirus pandemic, it has become apparent that children are less severely affected by an infection with the SARS-CoV-2 virus than adults [1, 2]. However, apart from acute symptoms experienced following an infection with the virus, the term post-COVID-19 or long COVID-19 was introduced, describing a broad variety of persisting symptoms following the infection with the virus in adults and children [3, 4]. These long-term symptoms seem to be indifferent to the clinical course of the initial infection and can lead to severe impairment of daily activities and quality of life [4].

Several studies have investigated this phenomenon in the pediatric population. Post-acute sequelae of COVID-19 (PASC) in children seem to manifest mainly after mild courses of COVID-19 [5]. Frequent symptoms include fatigue, exercise intolerance, and anxiety [5]. Especially children with a history of allergic diseases, obesity, or multisystem involvement seem to be at a higher risk for developing PASC [6].

Few studies have used cardiopulmonary exercise testing (CPET) for objectifying the subjective exercise intolerance observed by children suffering from PASC [7–9]. All studies in children reported an impaired functional capacity expressed by a low peak oxygen uptake ($\dot{V}{O}_{2}peak$) [7–10]. They also show a pathological minute ventilation in relation to carbon dioxide elimination slope (${\dot{V}}_{E}/{\dot{V}}_{CO2}$-slope) which is indicative of ventilatory inefficiency [7] along with a reduced oxygen uptake slope (${\dot{V}}_{O2}/P$-slope) which could be a sign of muscle deconditioning [7]. However, cardiac parameters like heart rate and O_2_ pulse (surrogate parameter for cardiac output) were comparable between children suffering from PASC compared to age-matched controls [11], as were pulmonary parameters like breathing reserve (BR) and minute ventilation (${\dot{V}}_{E}$) [7, 8].

Comparable findings have been described in adults. In a study by Dotan et al. [12], individuals with exertional dyspnea following mild COVID-19 demonstrated normal cardiopulmonary function as assessed by CPET, with oxygen uptake and O_2_ pulse within or above predicted values. These results indicate preserved functional capacity despite ongoing subjective symptoms [12].

While physical deconditioning likely contributes to the initially reduced performance, studies in adults suggest that it alone does not fully account for exercise intolerance. Additional mechanisms, such as subtle pulmonary or autonomic dysfunction, may also be involved [13]. Furthermore, limitations are linked to chronotropic incompetence, muscle dysfunction, and abnormal oxygen utilization [14].

Longterm studies in adults show that cardiopulmonary abnormalities improve over time; persistent symptoms seem not to correlate with objective heart or lung function measures, suggesting that functional and autonomic factors are as well central to exercise intolerance in long COVID [10, 14, 15].

So far, no longitudinal approach using CPET has investigated the long-term cardiopulmonary function after suffering from PASC. To the best of our knowledge, this is the first prospective study reinvestigating children suffering from PASC 6 months after a first exercise test in comparison with an unaffected control group.

## Material and methods

This study was approved by the Ethics Committee of the University of Erlangen-Nuremberg, FRG (206_21B). All study participants as well as their legal guardians gave written informed consent according to the standards set by the Declaration of Helsinki. The study is part of a larger study and was registered under clinicaltrials.gov as part of the FASCINATE study (NCT05445531) (https://clinicaltrials.gov/ct2/show/NCT05445531?term=FASCINATE&draw=2&rank=2).

### Participants

According to the protocol of the FASCINATE study**,** the children who had previously participated in the cross-sectional approach were reinvestigated 6 months after their initial examination [8].

At the time of the initial examination, these children were between 5 and 17 years of age. They were assigned to the PASC group based on the case definition provided in the German AWMF S1 guideline on long/post-COVID, which was in effect at the time of study initiation [16]. This definition is consistent with the World Health Organization (WHO) criteria for post-COVID-19 in children and adolescents. According to the WHO, post-COVID-19 is characterized by symptoms that begin within 3 months of a confirmed SARS-CoV-2 infection, persist for at least 2 months, and cannot be explained by an alternative diagnosis [17]. In addition, the condition must be associated with a limitation in everyday functioning. Within the broader category of post-acute sequelae of SARS-CoV-2 infection (PASC), post-COVID-19 thus represents a clinically defined subset. SARS-CoV-2 infection had also to be confirmed by polymerase chain reaction (PCR) testing. The comparison group consisted of children and teenagers with proof of SARS-CoV-2 infection by SARS-CoV-2-PCR**,** but PASC criteria [16] not fulfilled. All participants were required to be free of any acute infection at the time of each exercise test. We were able to reinvestigate 13 of the 20 children suffering from PASC and 23 of the 28 control children.

A stadiometer and electronic scale (Seca 704 S, Hamburg, Germany) were used to measure height and weight.

Symptoms corresponding with PASC were recorded at each test. Given the absence of significant differences in baseline pulmonary and cardiac function between groups, all participants were advised to gradually resume physical activity according to individual tolerance. This guidance aimed to support recovery of exercise capacity and prevent further deconditioning.

### Measurement of gas exchange

A small, low-dead-space respiratory valve (88 ml) with a size-matched mouthpiece and headgear was used (Metalyzer 3B, Cortex, Leipzig, Germany). Gas exchange was measured continuously using a breath-by-breath method and averaged over 15-s intervals during each test. For the completion of a valid $\dot{V}{O}_{2}peak$, two of the following physiological criteria must be met for validation: peak heart rate (peak HR) within 5% of the age-predicted maximum, volitional fatigue [18–21] or respiratory exchange ratio (RER) ≥ 1.0. A respiratory exchange ratio (RER) threshold of 1.0 was selected to define a valid V̇O_2_ peak, given that achieving higher RER values is more challenging in pediatric populations [21]. For children below the age of 8, the peak oxygen uptake was put in relation to normal values from Kalden et al. [22] and for children between 9 and 16 years of age to normal values from Bongers et al. [23]. Normal values for adults [24] were used for adolescents above the age of 16 years.

The ventilatory threshold (VT) was calculated by the *V*-slope method proposed by Beaver et al. [25], the ventilatory equivalent for oxygen uptake ($\dot{V}{E/\dot{V}O}_{2} and \dot{V}{E/\dot{V}CO}_{2}$), and the end-tidal pressure (petO_2_ and petCO_2_) method. OUES was determined by plotting oxygen uptake ($\dot{\mathrm{V}}{\mathrm{O}}_{2}$) against the logarithm of minute ventilation ($\dot{{V}_{\mathrm{E}}}$). The slope of this linear relation was calculated through single regression analysis [18]. The parameter $\dot{V}{O}_{2}$/*P*-slope describes the relationship between oxygen uptake ($\dot{V}{O}_{2}$) and external work rate (*P*) during incremental cardiopulmonary exercise testing [26].

A half-time recovery of $\dot{V}{O}_{2}$ (${T}_{1/2}\dot{V}{O}_{2}$) was defined as the time needed for $\dot{V}{O}_{2}peak$ to decrease by half [27] and was assessed during off transient after peak-graded CPET. Heart rate recovery was monitored during the first minute of the recovery phase (HRR).

### Cardiopulmonary exercise test

All subjects were assessed by using a 12-lead ECG (Custo®, Ottobrunn, Germany) for monitoring heart rate and ECG changes.

For cardiopulmonary exercise testing, an incremental step test was performed on a treadmill (COSMED T170, COSMED, Italy). We used an age-appropriate treadmill testing protocol resulting from a previous study [18]. We encouraged all participants verbally to run until exhaustion. The same researchers performed each test.

### Statistical analysis

Statistical analysis was performed using Microsoft Excel 2000® for data collection and SPSS 12.0® (SPSS Inc., Chicago, IL) for statistical evaluation. All measured values are reported as means and standard deviations. The Kolmogorov–Smirnov test was used to check for normal distribution. Homogeneity of variance was investigated using Levene’s *F*-test. For normally distributed variables, differences between the children suffering from PASC and the control group were assessed with unpaired *t*-tests; otherwise, the Wilcoxon or the Whitney-Mann *U* tests were used. For comparing the parameters at the two points in time, paired *t*-tests were used if the variables were normally distributed. Statistical significance was set at *p* < 0.05.

## Results

### Subjects

Overall, 13 of the previously investigated 20 participants suffering from PASC and 23 of the previously investigated 28 participants for the control group were retested 6 months after the initial assessment. There were no significant differences between the PASC group and the control group with respect to age, height, and weight (see Table 1). No significant differences in baseline cardiopulmonary exercise test (CPET) parameters were found between those of the PASC group who discontinued and those who completed the study (see Table 2). At the time of the second presentation, 3 of 13 PASC patients (23%) still reported symptoms (2 reported dyspnea and one cough). The amount of physical activity reported was comparable between the two groups. However, significant differences were observed in the duration of rest following SARS-CoV-2 infection and in subjective exercise tolerance post-infection (see Table 3).

**Table 1:** Characteristics of the participants as means ± standard deviation assessed with an unpaired *t*-test, as well as the *p*-values for each test (statistical significance was set at p < 0.05)

	PASC	Control	*p*-value
*n*	13	23	
Male (*n* (% of the respective group))	5 (38%)	12 (52%)	0.429
Female (*n* (% of the respective group))	8 (62%)	11 (48%)	0.429
Age (years)	13.6 ± 2.6	11.9 ± 3.1	0.113
Height (cm)	158.3 ± 13.8	157.0 ± 19.2	0.831
Weight (kg)	52.2 ± 14.2	48.4 ± 21.3	0.533
Body mass index (kg/m^2^)	20.7 ± 4.6	18.7 ± 4.6	0.225
Body surface area (m^2^)	1.5 ± 0.3	1.4 ± 0.4	0.601
Interval infection to initial CPET (days)	342.3 ± 240.2	315.2 ± 214.8	0.699
Interval infection to follow-up CPET (days)	585.6 ± 242.4	533.4 ± 216.5	0.524

Abbreviation: *PASC* post-acute sequelae of COVID-19

**Table 2:** Results within the PASC group between individuals who participated exclusively in the initial cardiopulmonary exercise test (PASC a) and those who completed both the first and second cardiopulmonary exercise test (PASC b) as means ± standard deviation assessed with an unpaired *t*-test, as well as the *p*-values for each test (statistical significance was set at p < 0.05) and Cohen’s *D* value for effect size

	PASC a	PASC b	*p*-value	Cohen’s *D* value
Peak RER	1.02 ± 0.09	1.05 ± 0.06	0.405	0.46
$\dot{V}{O}_{2}peak$(ml/kg/min)	34.3 ± 8.8	39.2 ± 8.7	0.264	0.56
$\dot{V}{O}_{2}peak$(% of normal value)	81.7 ± 24.9	91.0 ± 16.1	0.396	0.48
$\dot{V}{O}_{2}VT1$(ml/kg/min)	20.7 ± 6.0	22.8 ± 5.1	0.459	0.38
Peak velocity (km/h)	8.6 ± 3.0	10.5 ± 2.3	0.213	0.74
$\dot{V}Epeak$(l/min)	65.9 ± 27.3	74.0 ± 20.4	0.509	0.35
Peak BF (breaths/min)	58.1 ± 12.0	60.4 ± 12.0	0.696	0.19
Peak O_2_ pulse (ml)	9.6 ± 3.2	10.6 ± 3.2	0.515	0.32
Peak HR (beats/min)	179.3 ± 25.5	192.0 ± 7.0	0.241	0.79
HRR (beats/min)	− 17.4 ± 4.8	− 18.9 ± 7.1	0.594	− 2.3
Chronotropic index	73.3 ± 22.6	82.9 ± 10.0	0.322	0.62
$\dot{V}{O}_{2}/P$-slope	11.0 ± 5.8	11.5 ± 2.0	0.824	0.16
OUES	2.0 ± 0.8	2.2 ± 0.7	0.531	0.31
$\dot{V}{E/\dot{V}CO}_{2}$-slope	33.5 ± 2.0	34.2 ± 3.5	0.613	0.21
${T}_{1/2}\dot{V}{O}_{2}$(s)	110.0 ± 50.6	92.3 ± 30.3	0.455	− 0.47
Exercise time (s)	889.7 ± 124.2	837.3 ± 168.1	0.440	− 0.34

Abbreviations: *PASC* post-acute sequelae of COVID-19, *Peak RER* respiratory exchange ratio at maximal exertion, $\dot{V}{O}_{2}peak$ peak oxygen uptake, $\dot{V}{O}_{2}VT1$ $\dot{V}{O}_{2}$ at ventilatory threshold 1, $\dot{V}Epeak$ peak minute ventilation, *BF* breathing frequency, *BR* breathing reserve, *peak HR* peak heart rate, *HRR* heart rate recovery, *OUES* oxygen uptake efficiency slope, $\dot{V}E/\dot{V}C{O}_{2}$ breathing efficiency, ${T}_{1/2}\dot{V}{O}_{2}$ $\dot{\mathrm{V}}{\mathrm{O}}_{2}\mathrm{peak}$ decrease by half

**Table 3:** Physical activity profile and data from the spirometry of all participants as means ± standard deviation assessed with an unpaired *t*-test (* identifies a statistical significance set at *p* < 0.05), as well as the *p*-values for each test

	PASC	Control	*p*-value
Physical activity before pandemic (h/week)	3.6 ± 2.0	3.3 ± 3.1	0.763
Physical activity during pandemic (h/week)	1.6 ± 2.1	2.3 ± 3.1	0.474
Physical activity before infection with SARS-CoV-2 (h/week)	1.9 ± 2.3	2.7 ± 3.2	0.489
Duration of rest from physical activity (weeks)	4.1 ± 6.6	0.2 ± 0.7	0.024*
Worse subjective exercise tolerance after infection with SARS-CoV-2 (*n*, %)	7 (54%)	2 (9%)	0.003*

Abbreviation: *PASC* post-acute sequelae of COVID-19

### Cardiopulmonary exercise test

The data from the first and second cardiopulmonary exercise tests are summarized in Tables 4 and 5.

**Table 4:** Results from the first and second cardiopulmonary exercise tests of the whole test subjects as means ± standard deviation assessed with a paired *t*-test (* identifies a statistical significance set at *p* < 0.05), as well as the *p*-values for each test and Cohen’s *D* value for effect size

	Initial test	Control test	*p*-value	Cohen’s *D* value
Peak RER	1.06 ± 0.05	1.05 ± 0.07	0.471	0.12
$\dot{V}{O}_{2}peak$(ml/kg/min)	41.9 ± 7.7	44.1 ± 8.8	0.017*	− 0.42
$\dot{V}{O}_{2}peak$ (% of normal value)	93.1 ± 18.1	98.1 ± 22.5	0.170	− 0.23
$\dot{V}{O}_{2}VT1$(ml/kg/min)	24.2 ± 4.9	23.9 ± 7.1	0.468	0.04
Peak velocity (km/h)	10.7 ± 2.3	10.8 ± 2.2	0.468	− 0.12
$\dot{V}Epeak$(l/min)	77.0 ± 28.4	80.9 ± 31.6	0.116	− 0.27
Peak BF (breaths/min)	61.9 ± 12.4	59.7 ± 11.5	0.191	0.23
Peak O_2_ pulse (ml)	11.0 ± 4.2	12.0 ± 4.7	0.003*	− 0.53
Peak HR (beats/min)	191.6 ± 9.1	192.1 ± 10.2	0.768	− 0.05
Peak HR (% of normal value)	98.0 ± 5.7	97.5 ± 7.1	0.531	0.45
HRR (beats/min)	− 19.7 ± 5.0	− 19.1 ± 5.7	0.557	− 0.09
Chronotropic index	83.6 ± 9.9	84.1 ± 13.4	0.749	− 0.05
$\dot{V}{O}_{2}/P$-slope	12.9 ± 3.4	14.0 ± 4.9	0.174	− 0.23
OUES	2.4 ± 1.1	2.3 ± 1.1	0.971	0.06
$\dot{V}{E/\dot{V}CO}_{2}$-slope	34.6 ± 5.1	31.6 ± 8.3	0.020*	0.41
${T}_{1/2}\dot{V}{O}_{2}$(s)	85.3 ± 30.7	105.7 ± 32.4	0.015*	− 0.43
Exercise time (s)	926.2 ± 197.0	839.2 ± 273.2	0.055	0.65

Abbreviations: *Peak RER* respiratory exchange ratio at maximal exertion, $\dot{V}{O}_{2}peak$ peak oxygen uptake, $\dot{V}{O}_{2}VT1$ $\dot{V}{O}_{2}$ at ventilatory threshold 1, $\dot{V}Epeak$ peak minute ventilation, *BF* breathing frequency, *BR* breathing reserve, *peak HR* peak heart rate, *HRR* heart rate recovery, *OUES* oxygen uptake efficiency slope, $\dot{V}E/\dot{V}C{O}_{2}$ breathing efficiency, ${T}_{1/2}\dot{V}{O}_{2}$ $\dot{\mathrm{V}}{\mathrm{O}}_{2}\mathrm{peak}$ decrease by half

**Table 5:** Results from the first and second cardiopulmonary exercise tests as means ± standard deviation assessed with an unpaired *t*-test (* identifies a statistical significance set at *p* < 0.05), as well as the *p*-values for each test

	Test	PASC	Control	*p*-value	Cohen’s *D* value
Peak RER	Initial Follow-up	1.04 ± 0.07 1.05 ± 0.07	1.07 ± 0.05 1.05 ± 0.06	0.102 0.731	− 0.49 − 0.13
$\dot{V}{O}_{2}peak$(ml/kg/min)	Initial Follow-up	37.4 ± 8.8 39.5 ± 11.0	43.0 ± 6.7 45.5 ± 8.4	0.019* 0.101	− 0.74 − 0.64
$\dot{V}{O}_{2}peak$ (% of normal value)	Initial Follow-up	87.8 ± 19.5 96.5 ± 22.7	97.0 ± 19.3 99.0 ± 23.0	0.112 0.756	− 0.48 − 0.11
$\dot{V}{O}_{2}VT1$(ml/kg/min)	Initial Follow-up	22.1 ± 5.4 22.3 ± 7.6	24.7 ± 4.5 24.8 ± 6.8	0.079 0.330	− 0.55 − 0.36
Peak velocity (km/h)	Initial Follow-up	9.9 ± 2.6 10.5 ± 2.5	10.9 ± 2.4 11.1 ± 2.0	0.171 0.46	− 0.39 − 0.28
$\dot{V}Epeak$(l/min)	Initial Follow-up	71.0 ± 22.8 77.0 ± 33.0	78.0 ± 31.6 80.7 ± 32.7	0.411 0.746	− 0.22 − 0.11
Peak BF (breaths/min)	Initial Follow-up	59.6 ± 11.7 60.0 ± 10.3	64.3 ± 14.4 59.7 ± 12.3	0.244 0.940	− 0.34 0.03
Peak O_2_ pulse (ml)	Initial Follow-up	10.2 ± 3.2 11.4 ± 4.1	11.0 ± 4.5 12.1 ± 5.2	0.510 0.659	− 0.17 − 0.15
Peak HR (beats/min)	Initial Follow-up	187.3 ± 16.9 188.1 ± 15.5	190.5 ± 10.3 192.4 ± 10.6	0.434 0.384	− 0.25 − 0.34
Peak HR (% of normal value)	Initial Follow-up	96.1 ± 9.0 97.2 ± 9.1	97.8 ± 5.5 98.0 ± 5.9	0.442 0.395	− 0.25 − 0.11
HRR (beats/min)	Initial Follow-up	− 18.5 ± 6.4 − 16.8 ± 8.0	− 20.1 ± 3.8 − 20.4 ± 3.3	0.263 0.145	0.92 0.66
Chronotropic index	Initial Follow-up	79.6 ± 15.7 81.0 ± 16.1	83.8 ± 9.2 85.9 ± 11.5	0.287 0.348	− 0.35 − 0.37
$\dot{V}{O}_{2}/P$-slope	Initial Follow-up	11.4 ± 3.5 12.0 ± 6.2	13.9 ± 4.9 15.1 ± 3.6	0.057 0.124	− 0.58 − 0.65
OUES	Initial Follow-up	2.2 ± 0.7 2.0 ± 1.1	2.3 ± 1.2 2.6 ± 1.1	0.514 0.130	− 0.19 − 0.55
$\dot{V}{E/\dot{V}CO}_{2}$-slope	Initial Follow-up	34.0 ± 3.1 28.8 ± 6.2	35.2 ± 6.0 33.1 ± 3.6	0.426 0.246	− 0.24 − 0.53
${T}_{1/2}\dot{V}{O}_{2}$(s)	Initial Follow-up	97.9 ± 37.3 103.2 ± 36.5	82.5 ± 34.6 107.1 ± 30.5	0.154 0.750	− 0.43 − 0.12
Exercise time (s)	Initial Follow-up	855.6 ± 152.9 835.2 ± 196.1	969.9 ± 186.7 878.0 ± 259.9	0.025* 0.582	− 0.66 − 0.18

Abbreviations: *PASC* post-acute sequelae of COVID-19, *Peak RER* respiratory exchange ratio at maximal exertion, $\dot{V}{O}_{2}peak$ peak oxygen uptake, $\dot{V}{O}_{2}VT1$ $\dot{V}{O}_{2}$ at ventilatory threshold 1, $\dot{V}Epeak$ peak minute ventilation, *BF* breathing frequency, *BR* breathing reserve, *peak HR* peak heart rate, *HRR* heart rate recovery, *OUES* oxygen uptake efficiency slope, $\dot{V}E/\dot{V}C{O}_{2}$ breathing efficiency, ${T}_{1/2}\dot{V}{O}_{2}$ $\dot{\mathrm{V}}{\mathrm{O}}_{2}\mathrm{peak}$ decrease by half

**Fig. 1:**
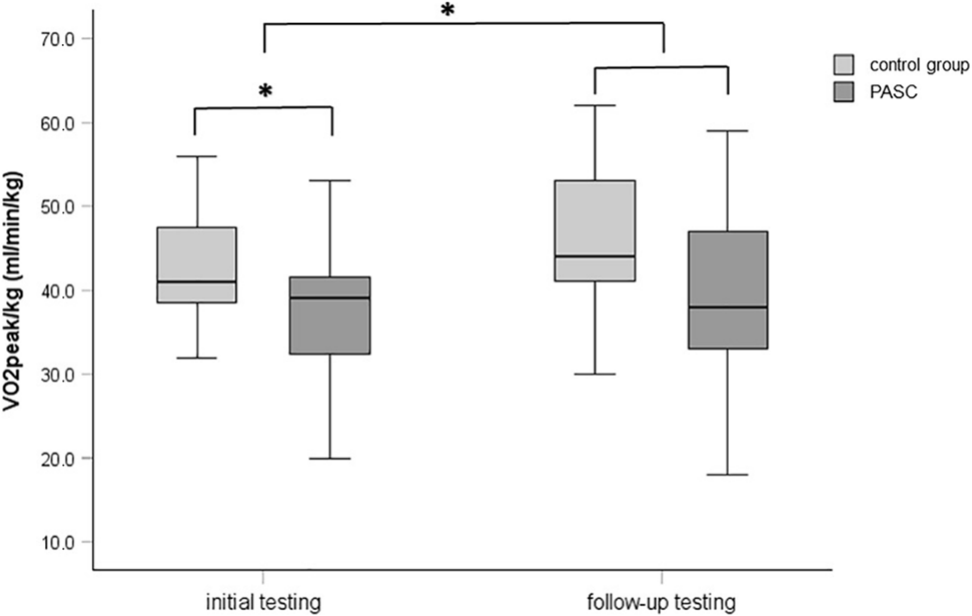
Median, as well as interquartile range, minimum and maximum of the $\dot{V}{O}_{2}peak$ between the children suffering from PASC and the control group between the first and second CPET, 6 months apart. Asterisk (*) identifies a statistical significance set at *p* < 0.05. Abbreviations: PASC, post-acute sequelae of COVID-19; $\dot{V}{O}_{2}peak$, peak oxygen uptake

At the initial test, the exercise time was significantly lower in the PASC group compared to controls. At follow-up, the exercise time had improved in the PASC group, and the difference between groups was no longer statistically significant, although values remained slightly lower. The previously observed difference of oxygen uptake ($\dot{V}{O}_{2}peak$) between the two groups could no longer be observed after a further 6-month interval. This was reflected in the significantly improved $\dot{V}{O}_{2}peak$ after 6 months in the overall group (see Fig. 1). Interestingly, this initial significant difference in $\dot{V}{O}_{2}peak$ did not reach significance in the PASC group nor in the control group on its own after 6 months but only when comparing both groups taken together after 6 months.

### Pulmonary function

**Fig. 2:**
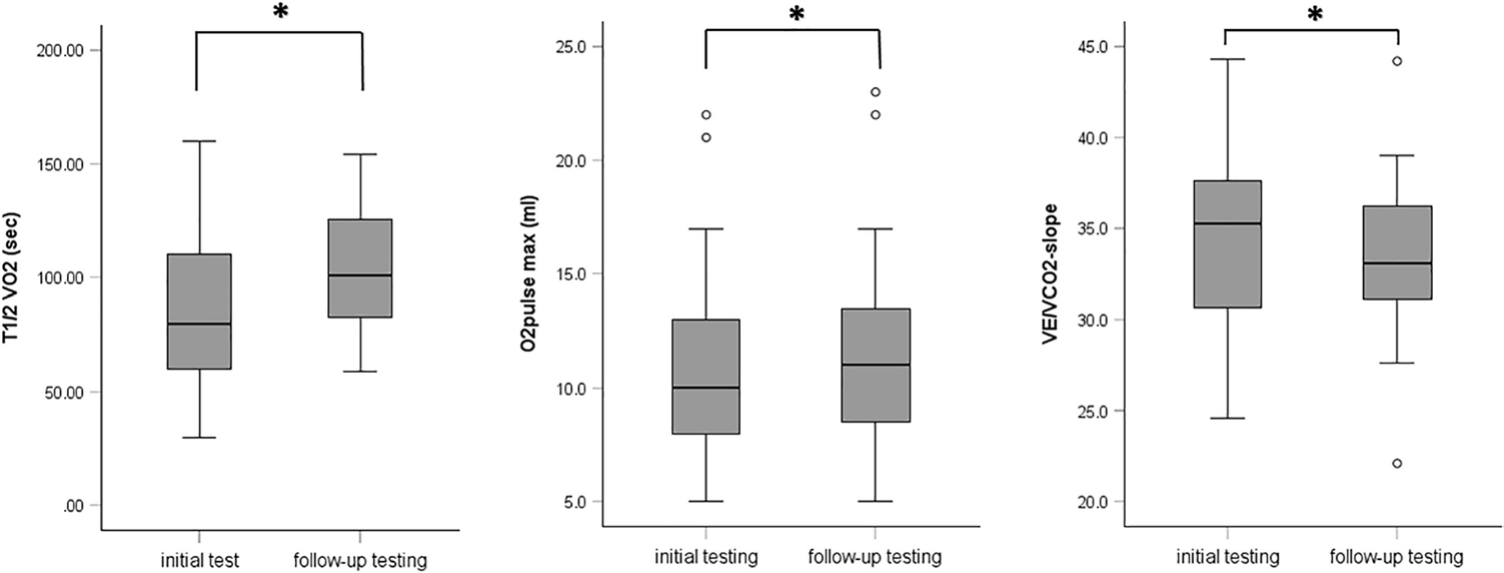
Median, as well as interquartile range, minimum and maximum of the O_2_ pulse max, $\dot{V}{E/\dot{V}CO}_{2}-\mathrm{slope}$ and ${T}_{1/2}\dot{V}{O}_{2}$ of the whole test group between the first and second CPET, 6 months apart. Asterisk (*) identifies a statistical significance set at *p* < 0.05. Abbreviations: $\dot{V}E/\dot{V}C{O}_{2}$, breathing efficiency; ${T}_{1/2}\dot{V}{O}_{2}$, $\dot{\mathrm{V}}{\mathrm{O}}_{2}\mathrm{peak}$ decrease by half

No pulmonary parameter from the CPET (minute ventilation either at peak exercise $\dot{V}Epeak$ or at the first ventilatory threshold $\dot{V}EVT1$ or breathing reserve, BR) showed a significant difference between the two groups at the first examination nor after 6 months (see Table 5). Nor was there an increase of pulmonary parameters over the course of the study. However, the $\dot{V}{E/\dot{V}CO}_{2}$-slope had decreased significantly when looking at the whole group consisting of the PASC children and the controls (see Fig. 2) after 6 months. When looking at the subgroups, this decrease of the $\dot{V}{E/\dot{V}CO}_{2}$-slope in neither group reached significance. Baldi et al. [7] combined the parameter of the $\dot{V}{E/\dot{V}CO}_{2}$ and petCO_2_ measured at the ventilatory threshold for differentiating signs of pulmonary artery hypertension. In this study, this parameter proved to be comparable between the PASC group and their controls. However, the parameter improved significantly after 6 months (see Table 4).

### Cardiac function

All parameters related to cardiac function (peak O_2_ pulse, peak HR, HRR, $\dot{V}{E/\dot{V}CO}_{2}$-slope, chronotropic index) were comparable between the two groups (see Table 5), both at baseline and after 6 months. However, a typical cardiac parameter, the O_2_ pulse, improved significantly over the course of 6 months when looking at the whole group, but when comparing the subgroups at baseline and after 6 months, this improvement did not reach significance (see Tables 4 and 5). However, the $\dot{V}{O}_{2}/P$-slope improved significantly in the control group but not in the PASC group.

### Peripheral function

The third physiologic compartment representing the mitochondrial function of the muscle [28] can be investigated using the time needed for the $\dot{V}{O}_{2}peak$ to decrease by half (${T}_{1/2}\dot{V}{O}_{2}$). This parameter was comparable between the two groups at baseline and after 6 months but had increased significantly after 6 months when both groups were looked at together (see Fig. 2 and Table 5).

## Discussion

### Physical activity, exercise time, and peak oxygen uptake

To the best of our knowledge, this is the first study reexamining children with PASC 6 months after the initial evaluation using an objectifiable method for the subjective symptom of exercise intolerance, namely cardiopulmonary exercise testing.

In a previous study, we were able to show that children suffering from PASC indeed exhibit reduced cardiopulmonary function, reflected by a significantly lower $\dot{V}{O}_{2}peak$ than a control group of children without symptoms of PASC [8]. These results have also been reported by other authors with a significantly reduced $\dot{V}{O}_{2}peak$ compared to healthy children [7, 9]. As part of the FASCINATE study, a second CPET was planned 6 months after the initial presentation. Interestingly, the previously observed impairment of cardiopulmonary function could not be objectified during the second CPET with comparable results between the two groups. Furthermore, both groups achieved comparable percentages of their predicted $\dot{V}{O}_{2}peak$ with 96.5% in the PASC group and 99% in the control group. This finding implies that children can recover from PASC and that at least for the subjects included in this study, 6 months seem to be long enough to recover from the disease. These findings align with previous studies in adult populations, which demonstrated a gradual recovery of cardiopulmonary function over time [14].

When comparing the measurements of $\dot{V}{O}_{2}peak$ from the second test with the first, it became apparent that all children improved their $\dot{V}{O}_{2}peak$ over the course of 6 months. Even though this improvement could not be seen within the subgroups (PASC and control), both groups showed an improvement that did not reach significance due to the relatively small sample size.

The fact that both groups improved their $\dot{V}{O}_{2}peak$ may still be a consequence of the detrimental effects of the lockdown during the COVID-19 pandemic when children could not participate in the recommended daily 60 min of physical activity [29]. A decline in cardiopulmonary function in children during and after the lockdown has been observed in several countries [30–34]. Children were spending more time at home with little access to structured activities [31, 32], leading to a decline in their cardiopulmonary function. Another explanation could be the possibility of both groups having suffered from an infection with SARS-CoV-2, and after 6 months, both groups showed an improvement.

As shown in adult studies, physical deconditioning likely contributed to the initially reduced cardiopulmonary performance; however, it alone does not fully explain persistent exercise intolerance in children with PASC, suggesting additional mechanisms such as subtle pulmonary or autonomic dysfunction [13].

The observed improvement of $\dot{V}{O}_{2}peak$ can be attributed to several of the three compartments described by Wasserman, namely the pulmonary, the cardiac, or the peripheral compartment.

### Pulmonary function

The fact that there were no significant differences between the pulmonary variables (${\dot{V}}_{E}peak$, ${\dot{V}}_{E}$ at VT1, breathing reserve BR, breathing frequency BF) recorded during CPET between the two groups was also observed at the second visit after 6 months. Although an overall improvement of the respiratory parameters could be observed after 6 months with an increase in ${\dot{V}}_{E}peak$ and a decrease in BF, this difference did not reach significance, nor was it limited to the children with PASC, but both groups showed this non-significant increase. Interestingly, although an infection with the SARS-CoV-2 virus mainly affects the lungs, the lungs seem not to be the cause of the reduced cardiopulmonary function in children suffering from PASC.

Another explanation for the reduced exercise capacity after suffering from COVID-19 is dysfunctional breathing [35, 36]. Functional breathing can be evaluated using the ventilatory equivalent (${\dot{V}}_{E}/{\dot{V}}_{C{O}_{2}}$-slope) [37]. In our previous study, we could not objectify a significant difference between the children suffering from PASC and those who had only undergone an infection with the SARS-CoV-2 virus. After 6 months, both groups still presented with comparable values for this parameter. We also could not find abnormal breathing patterns as described in adults [36]. However, when taken together, the two groups showed a significant decrease in the ${\dot{V}}_{E}/{\dot{V}}_{C{O}_{2}}$-slope, representing an improvement of the ventilation/perfusion mismatch and hyperventilation syndrome previously described in COVID-19 survivors [38]. A previous study investigating children suffering from PASC described a new combination of parameters of the ${\dot{V}}_{E}/{\dot{V}}_{C{O}_{2}}$ and the petCO_2_ at the ventilatory threshold as an indicator of pulmonary hypertension as the underlying cause for an altered ${\dot{V}}_{E}/{\dot{V}}_{C{O}_{2}}$-slope [7]. In their study, 48% of the subjects had a suspicious phenotype. In our study, this combined parameter did not differ between groups at either the first or the second presentation. However, there was a significant improvement of the quotient of these two parameters. The idea that some form of pulmonary hypertension could be involved during the SARS-CoV-2 infection is plausible, particularly when considering that certain genetic variants increase susceptibility to pulmonary vascular complications in long COVID patients. These variants, which are linked to endothelial dysfunction, coagulation pathways, and inflammatory responses, have been associated with the development of pulmonary hypertension in adults [39]. Targeting this pathology especially in children suffering from PASC could represent a new therapeutic option but needs further investigations although, to the best of our knowledge, there is currently no echocardiographic study in children with post-COVID-19 that supports this hypothesis [11] contrary to, e.g., children with MIS-C or acute COVID-19 infection [40, 41].

### Cardiac function

The most important parameters for unmasking cardiovascular limitations using CPET are the O_2_ pulse, the peak HR, and an abnormal increase of the $\dot{V}{O}_{2}/P$-slope. Most studies, including the investigation at the primary presentation of this study, [42] have shown normal values for the O_2_ pulse in patients suffering from post-COVID-19 [43] or recovering from severe illness [44]. However, there was a significant improvement of the O_2_ pulse in this study. This could be attributed to an improvement of cardiac function or a consequence of the previously described attribution of the reduced exercise capacity to pulmonary hypertension.

Similar findings were reported in adults after mild COVID-19, where O_2_ pulse and peak VO_2_ were normal or even slightly elevated compared to controls, suggesting no persistent cardiac impairment in this subgroup [12].

One cardiac cause for exercise intolerance in adult PASC patients is cardiac autonomic dysregulation which can be observed as chronotropic incompetence or inadequate heart rate recovery (HRR) [44]. The chronotropic incompetence is believed to be caused by SARS-CoV-2 infection of neurons and endothelial cells, chronic inflammation, or autoimmune mechanisms. In this study, neither the peak HR nor the HRR changed from the first study 6 months prior, nor was there any difference between the two groups, as observed during the earlier testing [8]. These results have also been observed in other studies using CPET for objectifying exercise intolerance in children suffering from PASC [7]. Possible reasons for these differences between adults and children could lie in the willingness to participate during CPET, reaching higher values, or in the more widespread use of beta-blockers in the adult population [8].

In addition, the generally normal BMI and non-smoking status in children may contribute to their more favorable recovery, as both obesity and smoking have been associated with a higher risk of persistent post-COVID-19 symptoms and reduced exercise capacity in adults [45].

A final parameter describing cardiac function is the $\dot{V}{O}_{2}/P$-slope reflecting limitations in the supply and/or metabolism of oxygen. None of the children investigated in this study exhibited pathological values, defined as values below 10 ml/min/W [46], nor was there a difference when compared to the control group. Most importantly, there was no further improvement of this parameter after 6 months.

### Peripheral function

Muscular and/or peripheral oxygen extraction abnormalities are believed to be in part responsible for the reduced exercise capacity observed in adults [14, 38]. Using noninvasive CPET for differentiating between deconditioning and altered oxygen delivery, mitochondrial dysfunction and muscular pathology, is difficult [7]. Most parameters like an abnormal response in the respiratory exchange ratio (RER), abnormal ${\overset.V}_{CO_2}$ -kinetics, a pathologic ${\overset.V}_{O_2}/P$-slope, or a reduced VT1 are unspecific and allow no differentiation to the cardiac component [37]. As described previously [42], none of these parameters showed any difference between the two groups, nor was there an improvement after a further 6 months.

## Conclusion

This prospective study was able to show that a reduced exercise capacity previously documented in children suffering from PASC can vanish within the timeframe of 6 months leading to comparable cardiopulmonary function with a healthy control group. Interestingly, the most significant improvements—apart from an increase in peak oxygen uptake capacity ($\dot{V}{O}_{2}peak$)—were observed in the perfusion-diffusion mismatch, as indicated by the ${\dot{V}}_{E}/{\dot{V}}_{C{O}_{2}}$-slope, and in cardiac stroke volume and, by extension, cardiac output, as reflected by peak O_2_ pulse during exercise. Both parameters are often used for detecting pulmonary artery hypertension. A further parameter pointing in this direction as a potential culprit for the experienced symptoms in children suffering from PASC is the combination of the ${\dot{V}}_{E}/{\dot{V}}_{C{O}_{2}}$ with the petCO_2_ at the ventilatory threshold. However, these parameters remained within normal limits, and consequently, pulmonary arterial hypertension is unlikely to have caused the initial symptoms.

## Limitations

The study has several limitations. Firstly, the sample size was relatively small, with only 13 children remaining in the post-COVID-19 group—a dropout rate of nearly 30%. This may introduce attrition bias and limit the generalizability of the findings. However, this concern is partly mitigated by the absence of significant differences in baseline cardiopulmonary exercise test (CPET) parameters between those who dropped out and those who completed the study. The high dropout rate likely reflects the demanding nature of the study protocol, which included a lengthy MRI and blood sampling, potentially discouraging follow-up participation. Furthermore, we did not include the socioeconomic background which may have indicated the accessibility to sports following the COVID-19 lockdown [47]. However, this study represents the first prospective study examining children suffering from PASC using CPET.

## Data Availability

No datasets were generated or analysed during the current study.

## Abbreviations

AWMF S1: Arbeitsgemeinschaft der Wissenschaftlichen Medizinischen Fachgesellschaften step 1
BF: Breathing frequency
BR: Breathing reserve
CPET: Cardiopulmonary exercise test
HR: Heart rate
HRR: Heart rate recovery
OUES: Oxygen uptake efficiency slope
PASC: Post-acute sequelae of COVID-19
Peak HR: Peak heart rate
peakRER: RER at maximal exertion
petO_2_: Partial pressure of end-tidal O_2_
petCO_2_: Partial pressure of end-tidal CO_2_
RER: Respiratory exchange ratio
${T}_{1/2}\dot{V}{O}_{2}$: $\dot{V}{O}_{2}peak$ Decrease by half
$\dot{\mathrm{V}}C{O}_{2}$: Carbon dioxide elimination
$$\dot{V}E$$: Minute ventilation
$\dot{V}Epeak$: Peak minute ventilation
$\dot{V}E/\dot{V}C{O}_{2}$: Minute ventilation in relation to carbon dioxide elimination
$$\dot{V}{O}_{2}$$: Oxygen uptake
$$\dot{V}{O}_{2}peak$$: Oxygen uptake
vpeak: Peak speed
VT: Ventilatory threshold
